# Computer-Based Cognitive Training Improves Brain Functional Connectivity in the Attentional Networks: A Study With Primary School-Aged Children

**DOI:** 10.3389/fnbeh.2019.00247

**Published:** 2019-10-23

**Authors:** Noelia Sánchez-Pérez, Alberto Inuggi, Alejandro Castillo, Guillermo Campoy, Jose M. García-Santos, Carmen González-Salinas, Luis J. Fuentes

**Affiliations:** ^1^Department of Psychology and Sociology, University of Zaragoza, Teruel, Spain; ^2^Robotics Brain and Cognitive Sciences Unit, Center for Human Technologies, Istituto Italiano di Tecnologia, Genoa, Italy; ^3^Department of Basic Psychology and Methodology, Faculty of Psychology, University of Murcia, Murcia, Spain; ^4^Servicio de Radiología, Hospital Morales Meseguer, Murcia, Spain; ^5^Department of Developmental Psychology and Education, Faculty of Psychology, University of Murcia, Murcia, Spain

**Keywords:** brain functional connectivity, fMRI, computer-based training, attentional networks, inhibitory control, school-aged children

## Abstract

We have shown that a computer-based program that trains schoolchildren in cognitive tasks that mainly tap working memory (WM), implemented by teachers and integrated into school routine, improved cognitive and academic skills compared with an active control group. Concretely, improvements were observed in inhibition skills, non-verbal IQ, mathematics and reading skills. Here, we focus on a subsample from the overarching study who volunteered to be scanned using a resting state fMRI protocol before and 6-month after training. This sample reproduced the aforementioned behavioral effects, and brain functional connectivity changes were observed within the attentional networks (ATN), linked to improvements in inhibitory control. Findings showed stronger relationships between inhibitory control scores and functional connectivity in a right middle frontal gyrus (MFG) cluster in trained children compared to children from the control group. Seed-based analyses revealed that connectivity between the r-MFG and homolateral parietal and superior temporal areas were more strongly related to inhibitory control in trained children compared to the control group. These findings highlight the relevance of computer-based cognitive training, integrated in real-life school environments, in boosting cognitive/academic performance and brain functional connectivity.

## Introduction

Sánchez-Pérez et al. (2018) aimed to investigate whether computer-based cognitive training that combined tasks based on working memory (WM) and some mathematics tasks from a commercial company (for details, see Supplementary Material), could be effective to improve both cognitive and academic-related skills in school-aged children of primary education. Because the authors found that the main results were due to the training with WM tasks, in this study we will refer to it as WM-based training.

Improvement of cognitive skills was expected on the basis of previous evidence that revealed positive near transfer effects of WM-based training on visual (Thorell et al., 2009; Wong et al., 2014; Studer-Luethi et al., 2015) and verbal WM (Thorell et al., 2009; Wong et al., 2014), and intelligence (Jaeggi et al., 2008). Additionally, WM-based training has been associated with improvement in some academic-related outcomes, like math grades (Holmes and Gathercole, 2014), arithmetic (Bergman-Nutley and Klingberg, 2014), reading (Loosli et al., 2012; Karbach et al., 2015; Söderqvist and Bergman Nutley, 2015), and vocabulary (Studer-Luethi et al., 2015). On the other hand, mathematics training has also proved to improve children’s mathematics competencies (Dowker and Sigley, 2010; Holmes and Dowker, 2013) and academic achievements (Starkey et al., 2004; Bryant et al., 2008; Ehlert and Fritz, 2013; Holmes and Dowker, 2013). The studies targeting specific cognitive skills involved in EF (e.g., Rueda et al., 2005; Espinet et al., 2013; Bergman-Nutley and Klingberg, 2014; Gathercole et al., 2019) evidence the relevance of such kind of interventions in comparison with those based on a rather broad curricular approach (e.g., Bodrova and Leong, 2007; Traverso et al., 2015), because the implementation of the latter ones in school-based contexts often include methodological changes to the academic curricula, which may largely depend on education policy makers.

In contrast to most of previously mentioned studies, Sánchez-Pérez et al. (2018) computer-based training program was integrated into the school routine, being carried out under the supervision of designated teachers who had undergone a short training program. Because the children worked independently, the difficulty of the different activities comprising the program was adapted to each child’s ability and rhythm. These characteristics, rarely found in previous studies, might have brought about that both children and teachers thought of our program as an additional subject rather than an extra-scholar activity, fostering that far transfer effects involving inhibition skills, non-verbal IQ, mathematics and reading skills, were observed in trained children compared to children from the active control group.

Since attentional mechanisms are suggested to be the processes underlying the relation between WM and reasoning skills (Conway et al., 2003; Engle, 2018), as well as between WM and academic outcomes (Bergman-Nutley and Söderqvist, 2017), Sánchez-Pérez et al. (2018) argued that behavioral improvement observed in the trained children might rely on children’s abilities to control their attention.

Attention improvement has been observed in a variety of intervention approaches being one form of intervention called state training, which involves aerobic exercise or a variety of mindfulness techniques (for reviews, see Posner et al., 2015; Posner and Rothbart, 2018). However, most important for the current study is the form of intervention called network training. Network training comprises repetitions of attentional tasks that are supposed to tap the executive attentional network mainly (Rueda et al., 2005), or more complex tasks that require several forms of attention or executive functions, such as videogames or computer-based programs (Diamond and Lee, 2011; Diamond, 2016). In the current study, we used the latter form of network training (WM-based training) to boost attention, a cognitive control function crucial for children to success at school, which is thought to be greatly malleable at ages that range from childhood to adolescence (Rueda et al., 2004; see Zelazo and Carlson, 2012; Karbach and Unger, 2014, for reviews). Accordingly, here we just focused on the benefits of our WM-based training in brain functional connectivity that involves the attentional networks (ATNs). In addition, given the well-established co-occurrence of WM and inhibition, being both skills supporting each other (Diamond, 2013, 2016), we aimed to assess whether brain connectivity changes were also related to attention-dependent inhibition skills.

### WM and the Attention Networks

In studying WM brain regions, some authors have highlighted the involvement of the prefrontal cortex, superior parietal cortex, basal ganglia, and medial temporal lobe (Wager and Smith, 2003; Gazzaley et al., 2004; Collette et al., 2005; Axmacher et al., 2007; Koenigs et al., 2009; Eriksson et al., 2015), suggesting that WM emerges from the dynamic interaction of the prefrontal and parietal cortex, striatum, and medial temporal lobe (Gazzaley et al., 2004). In turn, these WM regions are involved in components of the attention networks (ATNs): the dorsal frontoparietal network (including the frontal eye field, the intraparietal sulcus, and the superior parietal lobe), and the ventral frontoparietal network (including the temporoparietal junction, the inferior frontal gyrus, and the middle frontal gyrus) (Corbetta and Shulman, 2002). Thus, aside the well-established relationship between WM and attentional control at the behavioral level (Engle, 2018), common brain areas between the two cognitive functions have also been established.

The aforementioned overlapping between brain areas involved in both WM and the ATNs, may explain previous results in which WM-based training induced brain activity changes in attention-related frontal and parietal cortices in adults (Hempel et al., 2004; Olesen et al., 2004; Klingberg, 2010; Takeuchi et al., 2010; Jolles et al., 2013; Thompson et al., 2016; Clark et al., 2017). However, these findings might not being applied to children’s brains, given that WM is notably under development during childhood (Olesen et al., 2004). In fact, the networks underlying WM tasks in adults have been reported as being less recruited or more immature during childhood and adolescence (Klingberg et al., 2002; Kwon et al., 2002; Ciesielski et al., 2006; Crone et al., 2006; Scherf et al., 2006). Importantly, WM-based training effects observed in children’s brain functional connectivity have not been commonly addressed, and the results have been found rather inconsistent. For instance, Jolles et al. (2013) found no effects in children’s brain functional connectivity after WM-based training, whereas increased functional connectivity between the right middle frontal gyrus (r-MFG) and frontoparietal areas was found in young adults. In contrast, Astle et al. (2015) observed an increased strength of children’s neural connectivity between frontoparietal networks, superior parietal cortex, and inferior temporal cortex, after performing WM-based training sessions at home. Although this last study suggests that such training induces changes in children’s brain connectivity, further studies are needed to identify the brain effects of WM-based training, mainly on inhibitory control. Note that inhibitory control has been thought to be essential to resist preponderant irrelevant responses, fostering the ability to focus and sustain students’ attention, and helping them to succeed at school.

### The Present Study

Parents and children involved in the Sánchez-Pérez et al. (2018) overarching study were invited to participate in a resting state fMRI study. A subsample of participants containing children from the training and control groups volunteered and provided informed consent to participate. They were scanned just before the beginning of the training period, concurrently with the administration of the pre-test tasks, and were scanned again approximately 6 months after the end of the training. First, we verified that the behavioral effects of training with the reduced fMRI sample were equivalent to those found with the entire sample. Second, we examined whether brain functional connectivity effects were induced by the WM-based training, specifically in the MFG area of the attentional networks and the frontoparietal network (Astle et al., 2015), guided by the results of previous related studies (Jolles et al., 2013; Astle et al., 2015). Finally, we investigated whether improvement in inhibition-related abilities after the training sessions were linked to specific changes in brain functional connectivity. As the majority of WM-based training effects can be accounted for in terms of attentional control, we investigated changes within the right and left ATNs, as they were expected to underlie WM mechanisms, as well as changes between some regions of the ATNs and other brain areas involved in attention-dependent inhibitory control.

## Materials and Methods

### Participants

Participants in this study were a subsample of those who took part in a study that analyzed the behavioral effects of a computer-based training program on primary school-aged children (for further details, see Sánchez-Pérez et al., 2018). The final sample for the functional connectivity study was composed of 33 children in the training group (19 boys, 14 girls; ages *M* = 9.06, *SD* = 1) and 23 children in the control group (15 boys, 8 girls; ages *M* = 9.22, *SD* = 1.31) (more details about the participants’ recruitment and exclusion criteria are shown in Supplementary Figure S1).

### Procedure

The study was approved by the Ethics Committee of the University of Murcia and it was conducted in accordance with the approved guidelines and the Declaration of Helsinki. Written informed consent was obtained from the parents that volunteered to participate, and verbal assent was obtained from each child prior to each session. The parents sent the completed consent forms back to the school, where they were collected by a research assistant. After the parental consents were obtained, the parents completed the socioeconomic questionnaires.

This study followed a longitudinal design with three phases (baseline, training, and follow-up), and both groups were required to complete pre- and post-assessments. In the baseline phase, we collected the behavioral data in approximately 3 weeks and the fMRI data in another 3 weeks. In the second phase (training), children from the training group practiced the math training exercises (first part of the training session) and WM tasks (second part of the training session) in two weekly 30-min sessions over 13 weeks (training group). While the training group was performing the training activities, the active control group was engaged in the standard educational exercises in the computer classroom for the same duration. In the follow-up phase, behavioral data were collected in the training and control groups 1 week following the end of the training, whereas the fMRI data were collected by 6 months later. Relevant details about the activities comprising the computer-based training program as well as the behavioral data collected from children are given in the SM (see Sánchez-Pérez et al., 2018 for a more complete information about both the activities of the training program and the behavioral results with the entire sample).

### Neuroimaging Acquisition

Children were instructed to lie as still as possible with their eyes closed and not focus on a specific activity or thought. To ensure the child’s comfort, a parent sat in silence next to the child in the room during the scanning session. Hearing was protected using earplugs, and motion was minimized using soft pads fitted over the ears.

Functional and anatomical MRI data were acquired in a GE 1.5 T HDX scanner. fMRI analysis was performed according to a consolidated pipeline (Agosta et al., 2014; Inuggi et al., 2014; Imperiale et al., 2018) using FSL software (FSL v5.0)^1^. For the resting state sequence, 200 echo-planar imaging (EPI) images sensitive to BOLD contrast were acquired in 6 min and 30 s with the following sequence parameters: 24 slices; repetition time (TR), 1.888 ms; echo time (TE), 55 ms; voxel size, 4 × 4 × 4 mm; FOV, 25.6 cm × 25.6 cm; 64 × 64 matrix; flip angle, 90°. A high-resolution T1-weighted scan was acquired using a 3D FSPGR BRAVO sequence to enable co-registration of the functional data to an anatomical template (see section “Group template and dual regression” for details). The sequence parameters were as follows: TR, 12.4 ms; TE, 5.2–15 ms; voxel size, 1 × 1 × 1 mm; flip angle, 12°; 142 axial slices. In addition, a one-volume EPI covering the whole brain (38 slices) with the same sequence acquisition parameters as those used for the resting state was recorded to improve the co-registration accuracy.

#### Pre-processing

Pre-processing was performed using FEAT (Smith et al., 2004) and included (i) removal of the first four volumes to allow for signal equilibration, (ii) head movement correction by volume realignment to the middle volume using MCFLIRT, (iii) global 4D mean intensity normalization, and (iv) spatial smoothing (5 mm FWHM). We then applied ICA-AROMA (Independent Component Analysis-based Automatic Removal Of Motion Artifacts; see Pruim et al., 2015) to identify independent components (ICs) representing motion-related artifacts. This method calculates a set of spatial and temporal discriminative features and accordingly exploits a classification procedure to identify ICs representing motion artifacts. Specifically, these features evaluate the spatial overlaps of each component with the brain and cerebral spinal fluid (CSF) edges, the frequency content and the temporal correlation with realignment parameters of the IC time series. Finally, ICs classified as motion-related were removed from the fMRI dataset by means of linear regression. The resulting fMRI dataset was then high-pass filtered (cut-off frequency of 0.01 Hz), and the mean values of the BOLD signal in both liquor and white matter were regressed from the data. EPI_CLEAN_INDIVIDUAL images were finally obtained.

#### Anatomical Group Template

Considering that using an adult-based anatomical template would have introduced a severe bias into the pediatric imaging data by introducing anatomical co-registration errors (Hoeksma et al., 2005), we created a custom pediatric template using a procedure previously used to analyze resting state data in children (de Bie et al., 2012; Inuggi et al., 2014). First, we registered each participant’s T1 image to the MNI152 T1 brain template using a 12-DOF affine transformation in FLIRT (FMRIB version 5.92, Oxford, United Kingdom). Then, the mean inverse transformation of all the participants was calculated and applied to the MNI152 template (4 mm isotropic resolution) to create the pediatric custom template of our children participants. Second, we co-registered the individual RS EPI images to the common template following a three-step procedure: (a) non-linear co-registration of the individual RS EPI images to their corresponding one-volume whole-brain EPI images, (b) co-registration of whole-brain EPI images to their corresponding T1 image using affine boundary-based registration as implemented in FLIRT (Greve and Fischl, 2009), and (c) non-linear co-registration of the individual T1 image to the pediatric template (resampled at 4 × 4 × 4 mm) using FNIRT (Andersson et al., 2007). Finally, the resulting multi-step co-registration matrix was then applied to the individual RS EPI images to obtain the EPI_CLEAN_TEMPLATE images.

#### Functional Connectivity Analyses

We first were interested in determining whether our training program induced exclusive functional connectivity changes (post-training minus pre-training) in the trained group, that were not observable in the control one. Then, we addressed whether these connectivity differences could be directly related to behavioral changes. According to what it is stated in the manuscript, we started our exploration from the two Attentional Networks (ATNs), and later on we extended our analysis to observe how sensitive regions within the ATNs altered their connectivity with the rest of the brain. This allowed us to go deep into the relationships between behavioral and connectivity changes.

#### Within-ATNs (MELODIC)

To assess within networks longitudinal differences in brain connectivity in children, EPI_CLEAN_TEMPLATE images for each session for each participant were temporally concatenated across participants to create a single 4D dataset of 112 images. This fMRI dataset was then decomposed into ICs with a free estimation of the number of components using MELODIC (Multivariate Exploratory Linear Optimized Decomposition into Independent Components (Beckmann et al., 2005). To identify the participant-specific temporal dynamics and spatial maps associated with each individuated RSN, a dual regression analysis was applied (Filippini et al., 2009). This method implies (i) the use of the selected group-IC spatial maps in a linear model fit (spatial regression) against the single participant fMRI dataset, resulting in matrices describing the temporal dynamics for each IC and participant, and (ii) the use of these time-course matrices, which are entered into a linear model fit (temporal regression) against the associated fMRI dataset to estimate participant-specific spatial maps. After the dual regression, spatial maps of all the participants were grouped into two single 4D files for each RSN of interest, containing: (a) all the baseline recordings; and (b) the algebraic difference of year one recordings minus the baseline recordings. Candidate RSNs of interest were selected by visual inspection based on previous literature (Smith et al., 2009).

Using the first 4D file (a) we assessed connectivity group differences at baseline, in order to exclude any bias originating from groups composition. Using the second 4D file (b) we evaluated longitudinal connectivity group differences to assess a general effect of our training program on these networks. We will refer to these analyses as *group-within-network (GWN) analysis*.

Then, to investigate whether behavioral changes in inhibition-related scores (namely children’s responses in the go/nogo type 1 condition) were directly related to connectivity differences within the two ATNs, we evaluated the interaction between the two groups and that brain connectivity score. We will refer to these analyses as *score-within-network (SWN) analysis*.

Within-RSN connectivity differences were carried out with non-parametric permutation tests (5000 permutations), and analyses were restricted within the spatial RSN of interest using binary masks obtained by thresholding the corresponding Z map image (Z > 2.3). Output maps were threshold-free cluster enhancement (TFCE) corrected using a significance threshold of *p* < 0.05. Details of the statistical model used were defined in a following paragraph.

#### Seed-Based Function Connectivity Analysis

To understand whether inhibition-related scores also modulated how regions resulting from the previous analysis connected with the rest of the brain, we performed a seed-based functional connectivity analysis evaluating again the group – by – inhibition-related score changes interaction on brain connectivity evolution. We will refer to these analyses as score-whole-brain (SWB) analysis.

At the participant level, the regions of interest (ROIs) in the previous analysis that were sensitive to the statistical contrasts of interest were co-registered to each participant’s EPI space using a 12 DOF linear affine transformation implemented in FLIRT, and their mean (across space) time series were then calculated. These time series were used as regressors in a single GLM (one for each RSN producing a significant cluster) to explore correlations between these time series and BOLD signal fluctuations in the EPI_CLEAN_INDIVIDUAL images. In the case of multiple clusters emerging from the melodic analysis of a single RSN, replicating previous studies investigating the functional connectivity of multiple brain ROIs (Di Martino et al., 2008; Inuggi et al., 2014), regressors were orthogonalized according to the Gram Schmidt process as implemented in FEAT. The outputs of this analysis are subject-level maps of all those voxels correlating and anti-correlating with each investigated the ROIs. This process was repeated for the 112 EPI_CLEAN_INDIVIDUAL images of the baseline and follow-up recordings. Longitudinal differences between these maps were obtained from a second level, fixed-effects FLAME analysis using a GLM of 112 rows, 56 columns and 56 contrasts. The latter outputs were then inputted in a third-level GLM analyses testing the M2 model (defined in the next paragraph) assessing the interaction between group and inhibition-related score, using age as nuisance variable. Such third-level group analyses were carried out using a mixed-effects (FLAME) model as implemented in FSL. Corrections for multiple comparisons were carried out at the cluster-level using Gaussian random field theory (*Z* > 2.3; cluster significance: *p* < 0.05, corrected).

### Statistical Models

Two general linear models (GLMs) were tested. The first model (M1) was used for GWN analysis and aimed to assess whether a functional connectivity pattern was present in each group and whether it differed between the two groups of participants. A preliminary evaluation of the age × group interaction was conducted. Since no significant results were found, age was inserted as simple nuisance regressor to correct data for its effect. This analysis was performed integrating the classical two-sample unpaired *t*-tests^2^ with two additional contrasts for each group ([1 0 0], [−1 0 0], [0 1 0] and [0 −1 0], being each column the control and the trained group and participants’ ages).

The second model (M2) was used for SWN and for the final step of SWB and assessed age-corrected interactions between the group factor and inhibition-related score, by evaluating group differences in the slope of the linear correlation between inhibition-related scores and functional connectivity^3^.

Importantly, while the former model assessed general group-connectivity differences through the aforementioned GWN analysis, the latter model provided a specific evaluation of the modulatory effects of inhibition-related scores over brain functional connectivity through the aforementioned SWN and SWB analyses. All the inhibition-related scores were calculated as follows: baseline values were subtracted from those collected at year one, and the values (including age) were then demeaned according to the whole-participant average. Inhibition-related score was calculated as follows: baseline values were subtracted from those collected at year one. Resulting values were then demeaned according to the whole-participant average. Participants’ age was also demeaned.

## Results

Behavioral results were conducted here just to assure that the main findings of Sánchez-Pérez et al. (2018) with the entire sample reproduced with the fMRI subsample. Significant differences were found in SES scores between families of both groups at baseline, whereas there were not significant results regarding the mothers’, fathers’, and children’s ages. The difference in SES between families from the two schools was an unexpected result. Those schools were chosen precisely because supposedly families matched in education level and incomes. However, it is important to note that in the time of testing, our country was immersed in an important economic crisis that impacted the rate of unemployment, reducing dramatically families’ monthly income. It might have affected families from one school more than families from the other school, producing the observed differences in the SES scores. It is likely that the observed differences in SES reflected a timely circumstance rather than a constant situation of the families, and then that the differences, mainly regarding family income, were artifactual. Nonetheless, we decided to include SES as a control variable.

Independent *t*-tests revealed significant gender effects on the go/nogo, and math fluency. Also, the ANOVA yielded significant differences in math grades and scores in standardized tests as a function of children’s age. Consequently, SES, gender, and age were included as control variables in further behavioral analyses.

ANCOVAs were conducted to examine the training efficacy on the children’s academic and cognitive performances between the training and control groups. As it was argued above, we used the pre-test scores for each task, SES, gender, and age as control variables. The results confirmed significant group effects on post-training scores in the following tasks: math fluency (*F*[1,49] = 5.14, *p* = 0.028, $\eta_{\text{p}}^{2}$ = 0.10); reading skills (*F*[1,49] = 5.53, *p* = 0.023, $\eta_{p}^{2}$ = 0.10); math grade (*F*[1,48] = 18.66, *p* < 0.0001, $\eta_{p}^{2}$ = 0.28); non-verbal IQ (*F*[1,49] = 5.75, *p* = 0.020, $\eta_{p}^{2}$ = 0.11); and percentage of errors on go/nogo task type 1 (*F*[1,46] = 4.18, *p* = 0.047, $\eta_{p}^{2}$ = 0.08), type 2 (*F*[1,46] = 8.69, *p* = 0.005, $\eta_{p}^{2}$ = 0.16), type 3 (*F*[1,46] = 8.07, *p* = 0.007, $\eta_{p}^{2}$ = 0.15), and total nogo (*F*[1,46] = 11.38, *p* = 0.002, $\eta_{p}^{2}$ = 0.20) (see Table 1). For these tasks, the analyses indicated that children engaged in the training group outperformed those in the control group.

**TABLE 1:** Training vs. control groups on pre- and post-training assessments: means, standard deviations, ANCOVA results (training vs. control groups), and effect sizes (partial eta squared).

		**Pre-training**		**Post-training**			**Group effect**	
**Task**	**Group**	**Mean**	***SD***	**Mean**	***SD***	***F***	**Direction**	**Effect size**
**WJ-III scores**								
Math fluency	Control	50.26	17.53	56.39	17.10	5.14^∗^	Training > control	0.10
	Training	46.06	12.61	59.85	14.81			
**PROLEC**								
Reading skills	Control	62.94	19.55	73.40	21.61	5.53^∗^	Training > control	0.10
	Training	63.64	17.21	79.65	21.08			
**School grades**								
Math grades	Control	2.18	1.33	1.64	1.43	18.66^∗∗∗^	Training > control	0.28
	Training	2.94	0.79	3.09	0.81			
**K-BIT**								
Non-verbal IQ	Control	96.87	13.03	96.30	12.07	5.75^∗^	Training > control	0.11
	Training	100.12	12.97	103.88	11.13			
**Go/nogo**								
Nogo (% errors)	Control	29.76	12.53	30.65	13.50	11.38^∗∗^	Control > training	0.20
	Training	33.48	18.60	18.85	10.86			
Type 1	Control	28.21	15.10	34.47	17.05	4.18^∗^	Control > training	0.08
	Training	36.80	21.14	22.29	16.54			
Type 2	Control	33.93	17.17	31.68	17.34	8.67^∗∗^	Control > training	0.16
	Training	31.39	20.20	20.56	13.33			
Type 3	Control	27.14	17.89	30.75	15.89	8.07^∗∗^	Control > training	0.15
	Training	29.87	18.85	19.48	13.63			

*^∗^*p* < 0.05; ^∗∗^*p* < 0.01; ^∗∗∗^*p* < 0.001.*

### Magnetic Resonance Imaging (MRI)

#### Within-ATNs Connectivity (The Group Within Network – GWN – Analysis)

**FIGURE 1:**
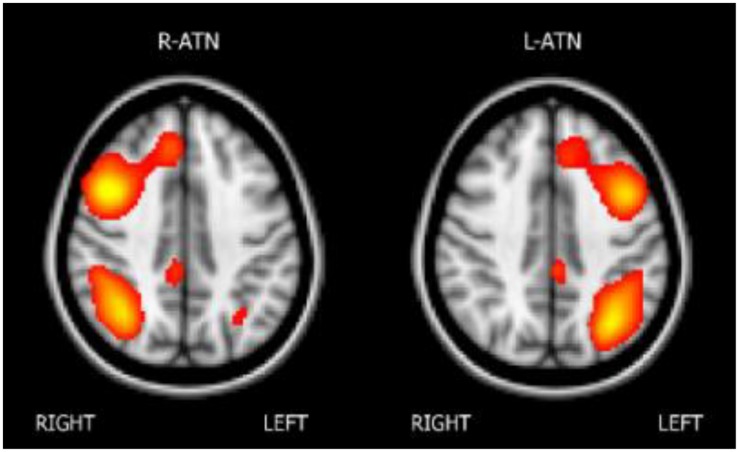
The attentional networks.

The melodic analysis reconstructed 41 ICs, among which twenty-two could be associated with a resting state network (RSN). We selected the attentional networks, as illustrated in Figure 1.

Considering that the group differences were calculated over both the right and the left ATNs, only voxels that showed a *p*-value below 0.025 after the TFCE correction were reported as significant (see Table 1). To exclude that longitudinal group differences might have emerged due to connectivity differences present at the beginning of the training, a preliminary melodic analysis with data at baseline was carried out within the two ATNs. The analysis did not reveal any significant differences in connectivity between the two groups.

**FIGURE 2:**
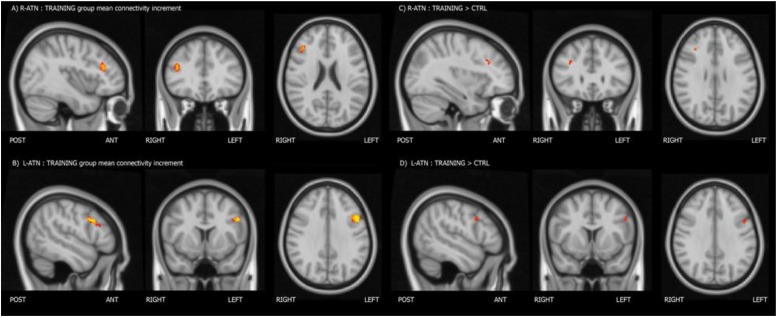
Mean connectivity increment within the training group **(A,B)**, and group-within-network (GWN) connectivity increment in the training group with respect to the control group **(C,D)**.

Melodic analysis of R-ATN revealed a cluster, located in the r-MFG, of increased longitudinal connectivity in the training group (Figure 2A), which was absent in the control group (not shown). When the connectivity profiles of the two groups were directly compared (Figure 2C), the training group showed a significant connectivity increment compared to the control one. The same result was symmetrically obtained in the left hemisphere investigating the L-ATN (Figures 2B,D). The results are summarized in Table 2.

**TABLE 2:** Significant differences between the training and control groups in the GWN analysis.

**RSN**	**Contrast**	**Stats (*p*/*t*-score)**	**Position (x,y,z) [mm]**	**Anatomical position**
R-ATN	TRAINING > CTRL	*p* = 0.020, *t* = 3.81	34, 30, 28	Right MFG
L-ATN	TRAINING > CTRL	*p* = 0.024, *t* = 3.74	−46, 10, 32	Left MFG

**FIGURE 3:**
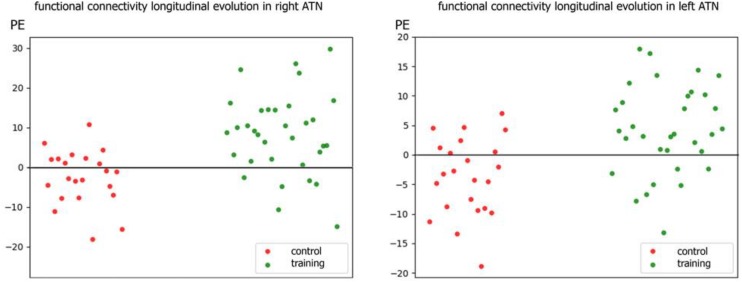
Scatter plot of individual parameters estimate (PE) of functional connectivity evolution in the training and control groups within R-ATN **(right)** and L-ATN **(left)**.

Individual PE (parameter estimate) values of the latter contrast showed that the increment in functional connectivity observed in the training group compared with the control group was not driven by extreme values (see Figure 3).

#### Inhibition Abilities

We tested children’s inhibitory control abilities through responses to nogo trials in the go/nogo task. Since in Durston et al.’s (2002) fMRI study, the difference in signal change was only statistically significant for the nogo after 1 preceding go trial type (i.e., type 1 condition), we assessed the interaction involving group and nogo scores taken children’s responses just in the go/nogo type 1 condition. For the sake of better legibility, we used participants’ hit rate in the nogo trials instead of the more standard children’s error rate (hits = 100 – errors). A hit occurred when the participant succeeded in withholding the response when a nogo trial was presented.

##### Group × nogo hits interactions within-ATNs connectivity (the Score Within Network – SWN – analysis)

**FIGURE 4:**
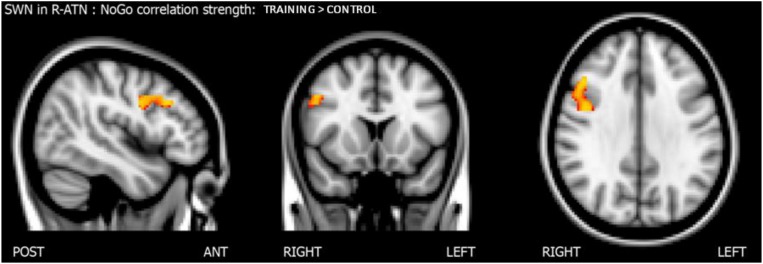
Results of the score-within-network (SWN) analysis within R-ATN: group × nogo hits interaction.

Considering that the interactions were calculated over both ATNs, only voxels that showed a *p*-value below 0.025 after the TFCE correction were reported as significant. Melodic analysis of R-ATN revealed that in a r-MFG cluster, the positive correlation between hits in nogo trials and functional connectivity had a higher slope in the training group compared to the control group (Figure 4). Thus, the increased connectivity within those brain areas were more strongly related to the increase of hits in nogo trials for children belonging to the training group than for children belonging to the control group. Results are summarized in Table 3.

**TABLE 3:** Summary of score-within-network (SWN) within R-ATN: *group* × nogo hits significant interaction.

**RSN**	**FC – nogo hits correlation slope**	**Stats (*p*/*t*-score)**	**Position (x,y,z) [mm]**	**Anatomical position**
R-ATN	TRAINING > CTRL	*p* = 0.006, *t* = 3.82	46, 22, 32	r-MFG
		*p* = 0.006, *t* = 3.80	42, 2, 32	Precentral gyrus

##### Group × nogo hits interactions across whole-brain functional connectivity of nogo-sensitive ROIs (the Score-Whole-Brain – SWB – analysis)

**FIGURE 5:**
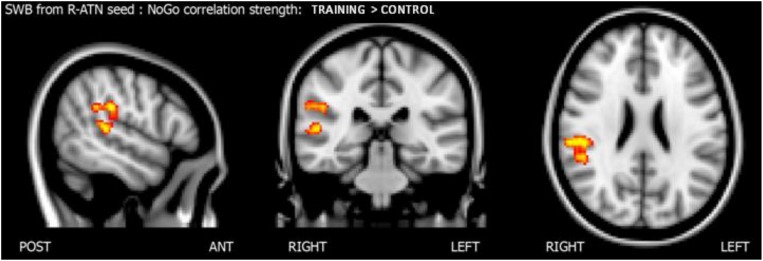
Score whole brain analysis. Results of the score-whole-brain (SWB) analysis from R-ATN seed: group × nogo hits significant interaction.

To understand how nogo-sensitive regions altered whole-brain connectivity as a function of the training program executed, we calculated the interaction between the latter regions’ seed based functional connectivity (SBFC) and nogo hits evolution. The interaction analysis revealed that the positive linear correlation between hits in the nogo condition and functional connectivity between the r-MFG cluster and the right parietal region (parietal operculum, supramarginal gyrus) had a higher slope in the training group compared to the control group, as shown in Figure 5. The results are summarized in Table 4.

**TABLE 4:** Summary of score-whole-brain (SWB) analysis from R-ATN seed: group × nogo hits significant interaction.

**Seed**	**Contrast**	**Stats (*p/t*-score)**	**Position (x,y,z) [mm]**	**Anatomical position**
R-ATN	TRAINING > CTRL	*p* = 0.009, *t* = 3374	52, −28, 26	Right parietal operculum/supramarginal gyrus
		*p* = 0.009, *t* = 3.53	52, −32, 8	Right superior temporal gyrus

## Discussion

We first assured that behavioral findings found with the entire sample reproduced with the fMRI subsample. As with the entire sample (see Sánchez-Pérez et al., 2018), the subsample of trained children showed significant improvement in cognitive and academic skills compared with children that served as the active control group. Beneficial effects were observed in non-verbal IQ, inhibitory control, math and language abilities, and math grades. Our results agree with previous studies showing that WM training has also resulted in children’s improvement in non-verbal IQ (Klingberg et al., 2002, 2005) and inhibition (Klingberg et al., 2005; Roughan and Hadwin, 2011), and in academic abilities (Holmes and Gathercole, 2014; Titz and Karbach, 2014; Blakey and Carroll, 2015; Söderqvist and Bergman Nutley, 2015; Studer-Luethi et al., 2015). The high correlation usually observed between WM and fluid intelligence may be due to the fact that both rely on the ability to control one’s attention. In other words, attention control appears to be the common mechanism underlying WM and intelligence (Engle, 2018). These attention control mechanisms would include the ability to maintain task-relevant information active (WM), and the versatility to engage relevant information and disengage from that when it is later proved to be wrong (intelligence). The inhibitory control relationship may be due to the need to inhibit distracting information for goal-directed behavior (Diamond, 2013). Concerning children’s academic improvements, WM likely underlies mental arithmetic and mathematics performance (DeStefano and LeFevre, 2004), whereas a WM component, the phonological storage, is believed to play an important role in the development of a variety of linguistic abilities, such as reading, vocabulary, and comprehension (Gathercole and Baddeley, 1990). WM is assumed to improve children’s abilities to control their attention, which may also affect their reading skills (Karbach et al., 2015). In summary, our WM-based training boosted WM-related processes, including cognitive and academic skills.

As Sánchez-Pérez et al. (2018) argued that attentional control processes may underlie benefits on both cognitive and academic outcomes, we just assessed brain functional connectivity changes in the attentional networks, linked to attention-dependent inhibitory control. We found a significant post-training difference between groups consisting of higher connectivity in ATN areas in the training group compared with the control group. This difference persisted even 6 months after the training was terminated, suggesting that the changes in functional connectivity last in time. Briefly, we observed an increased connectivity in the MFG, as well as important relationships between attention-dependent inhibitory control improvement and functional connectivity in a r-MFG cluster.

Trained children showed higher functional connectivity within the MFG, a region of the ATNs, compared to children who did not participate in the training. This substantially increase in connectivity in attentional areas after WM sessions is consistent with the connectivity results found in children (Astle et al., 2015) and young adults (Jolles et al., 2013). Altogether, the significant post-training effects on the ATNs support the notion that WM and attention are related processes in which attentional skills are required to actively keep relevant information in mind during simultaneous potent internal and external distractions (Unsworth et al., 2014).

Klingberg (2010) argued that WM-based training induces changes in a common neural network underlying WM and other cognitive skills. Our results suggest that, at least, part of this common network is the ATNs. The higher connectivity within the ATNs after training might also explain the aforementioned behavioral transfer effects. According to Constantinidis and Klingberg (2016), the transfer from a trained task to a non-trained task is likely associated with a shared reliance of those tasks on a frontoparietal network. Consequently, performing WM tasks would have boosted connectivity in the ATNs, which in turn might have enhanced children’s academic and cognitive performance.

Seed-based functional connectivity analyses also revealed that connectivity between the r-MFG and homolateral parietal and superior temporal areas was more strongly related to inhibitory control improvement in the training group than in the control group. The present results were consistent with prior ones in which the ability to inhibit preponderant responses was mainly lateralized in the right hemisphere (Garavan et al., 1999) and related to parietal areas (Durston et al., 2002). From a developmental perspective, previous studies have found that prefrontal areas and networks underlying WM in adults were less recruited or more immature during childhood and adolescence (Klingberg et al., 2002; Kwon et al., 2002; Ciesielski et al., 2006; Crone et al., 2006; Scherf et al., 2006). For instance, Mehnert et al. (2013) found that adults responded faster and more accurately than children in a go/nogo task, and importantly they also showed stronger frontoparietal brain activation in the former than in the latter group. However, as the frontoparietal network usually matures from childhood to adulthood, we should expect that any targeted intervention with tasks that involve that network, as well as other related attentional networks, should accelerate brain maturation. In line with this contention, Rueda et al. (2005) found effects after 5-day of executive attention training observing that children’s prefrontal ERP patterns resembled those exhibited by adults after the attention training. Interestingly, here we have found that children who performed the WM activities showed an increased functional connectivity in areas involved in these WM and attentional common networks, which resulted in a pattern of brain functional connectivity more similar to the adults’ findings. Therefore, our results indicate that WM training boosted school-aged children’s developmental changes in brain functional connectivity. Consequently, those children participating in the training group might have undergone enhanced brain maturation, rendering their connectivity patterns more similar to those of adults, resulting in increased inhibition abilities. We also observed a stronger relationship between inhibition improvement and higher connectivity between the r-MFG and right temporal areas, such as the superior temporal gyrus. This association is in line with the results of Rubia et al. (2007), in which adolescents’ probability of inhibition correlated with activation in the superior temporal gyrus.

Despite the open controversy regarding the benefits of WM-based training on children’s cognitive and academic skills, to our knowledge, few studies have addressed this question by analyzing the neuroimaging effects during childhood. In one of those studies, published by Jolles et al. (2013), children were required to practice a verbal WM task for 6 weeks 15 times, but no effects were found after the training. It is likely that increasing the length of the WM-based training, as we did in the current study, may help discerning brain functional connectivity changes in children, and hereby extend the benefits of training to cognitive and academic outcomes.

Although our findings shed light on the benefits of WM-based training on children’s academic and functional connectivity when applied in school settings, there are also some limitations. For instance, our fMRI follow-up measurements were collected approximately 6 months after the training sessions concluded; yet, taking the measurements also after 12 months is recommendable to observe long-term effects of the training. Additionally, our reduced sample did not allow analysis of potential age-related effects. Thus, further research should test whether age differences in functional connectivity patterns exist during childhood. Finally, convergent evidence by using alternative imaging techniques, such as EEG, is also desirable due to the limitation that oxygenation level-dependent (BOLD) measures convey concerning the origin of the recorded signals.

## Conclusion

This research contributed to the study of behavioral and functional connectivity benefits of WM-based training in school-aged children. Specifically, children of the training group were found to exhibit significantly improvement in attention-dependent inhibitory control, non-verbal IQ, mathematics and reading skills, compared to those in the active control group. More relevant, brain functional connectivity analyses showed that children undertaking the computer-based training exhibited higher connectivity in attentional brain areas after the sessions. A main characteristic of our training program that overcomes other previous interventions, is that it has been designed to be part of the school routine. The ease with which such a program can be implemented in real-life school environments, may have important positive consequences for academic success, not only for children with typical development, but also for children with special educational needs. Additionally, the simplicity of our computer-based training program, witnessed by the ease with which the involved teachers and students learned to manage it, suggests that with very few usability modifications, the program could be easily adapted to a remote utilization. Semi-automatic, computerized, remote training programs would represent a key strategy for national educational systems to increase the quantity and quality of their academic offer. Also, such a program could be carried out at students’ home environment, according to a flexible schedule, without the need of further supervision, and without increasing education costs. Altogether, this approach may thus contribute to reach a broader interest, including parents, teachers, and specially education policy makers. We hope that the present findings will have a notable impact in society, above all in those countries very much concerned with the high rate of their children that fail at school.

## Data Availability Statement

The research meta-data supporting this publication are available upon request to the senior author, LF.

## Ethics Statement

The studies involving human participants were reviewed and approved by the Comisión de Ética de Investigación, Universidad de Murcia. Written informed consent to participate in this study was provided by the participants’ legal guardian/next of kin.

## Author Contributions

LF conceived and designed the overarching study. LF, AI, and JG-S designed and implemented the MRI protocol. LF, AC, and GC designed the training tasks. AC adapted the training tasks to the videogame format and carried out the computer program. LF, NS-P, AI, GC, CG-S, and JG-S oversaw the testing and data collection. NS-P and CG-S conducted the behavioral analysis. AI conducted the neuroimaging analyses. NS-P, AI, and LF drafted the manuscript. LF, NS-P, AI, AC, GC, CG-S, and JG-S provided critical revisions of the manuscript.

## Conflict of Interest

The authors declare that the research was conducted in the absence of any commercial or financial relationships that could be construed as a potential conflict of interest.

## Footnotes

1 http://www.fmrib.ox.ac.uk/fsl

2 https://fsl.fmrib.ox.ac.uk/fsl/fslwiki/GLM#Two-Group_Difference_Adjusted_for_Covariate

3 https://fsl.fmrib.ox.ac.uk/fsl/fslwiki/GLM#Two_Groups_with_continuous_covariate_interaction
