# Supplementary material for: Computer-Based Cognitive Training Improves Brain Functional Connectivity in the Attentional Networks: A Study With Primary School-Aged Children
Source: Front Behav Neurosci. 2019 Oct 23;13:247. doi: 10.3389/fnbeh.2019.00247 (PMC6819316; doi:10.3389/fnbeh.2019.00247)
Supplement: Supplementary file 1 [file Data_Sheet_1.docx]

Supplementary Material

**Participants**

The first MRI data were obtained from 100 students (48 for the training group, 52 for the control group), whereas 71 students (41 for the training group, 30 for the control group) participated in the second MRI (follow-up phase). After excluding 15 children for excessive head movements during one of the two sessions, the remaining sample included 56 participants, 33 children in the training group (19 boys, 14 girls; ages *M* = 9.06, *SD* = 1) and 23 children in the control group (15 boys, 8 girls; ages *M* = 9.22, *SD* = 1.31). The diagram chart illustrating the recruitment of children for the fMRI study is shown in Figure S1. The descriptive statistics of the sample set are shown in Table S1.


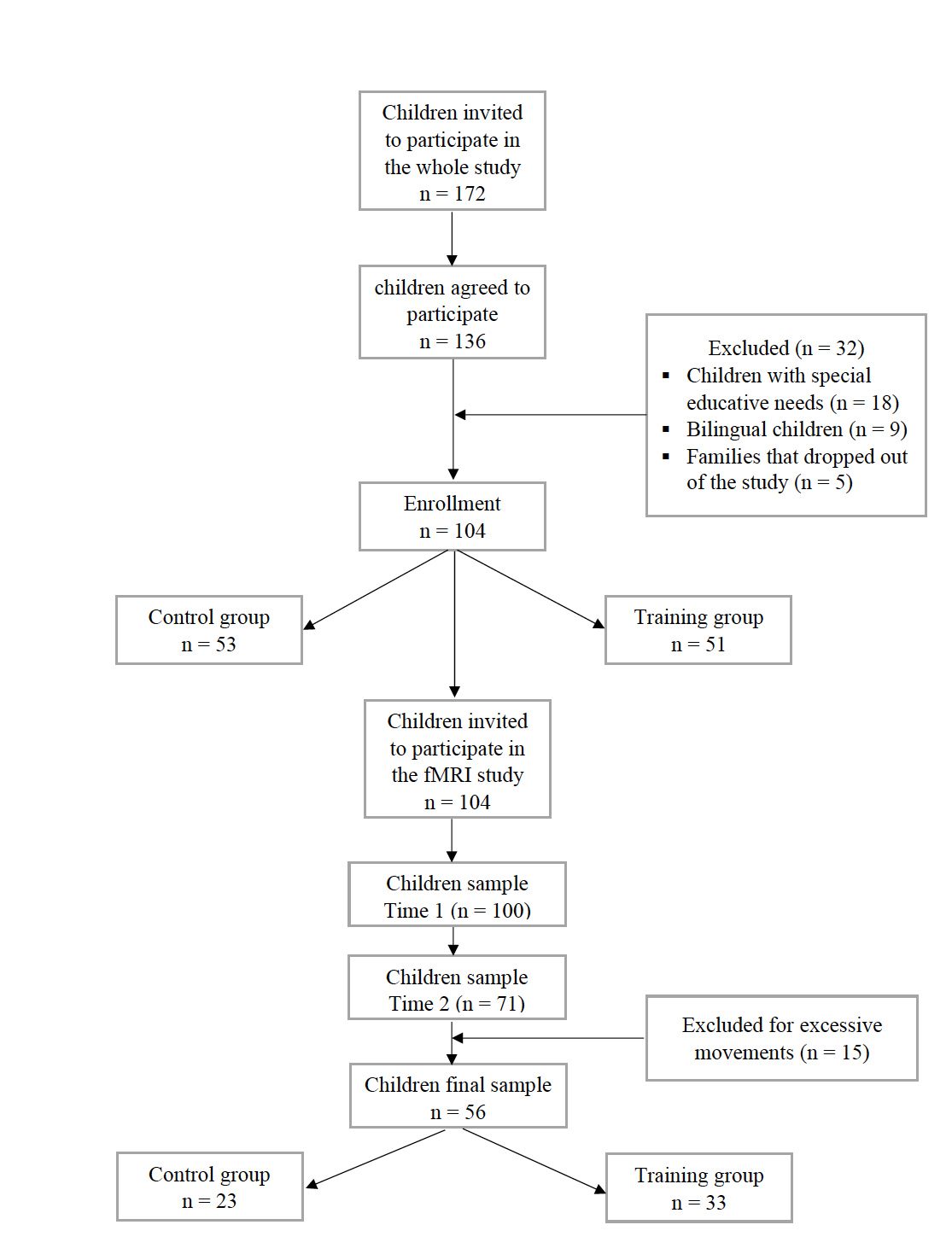


**Figure S1.** Diagram chart illustrating how the final subsample for the fMRI study was reached.

**Table S1. Means, standard deviations and comparison of families’ sociodemographic characteristics at baseline in the training and control groups.**

|  | Training group | Control group | *t*-test | P-value |
| --- | --- | --- | --- | --- |
| Mother’s age | 39.66 [5.18] | 40.33 [6.73] | *t* [51] = .41 | .681 |
| Father’s age | 41.43 [5.19] | 43.71 [5.59] | *t* [45] = 1.40 | .167 |
| Children’s age | 9.06 [1.00] | 9.22 [1.33] | *t* [54] = 0.51 | .614 |
| Family’s SES | .23 [0.77] | -.28 [0.90] | *t* [53] = -.2.21 | .032* |

* = *p* < .05

## Training Program

Three tasks were included in the WM-based training: (i) the *n*-back task, based on Pelegrina et al, (Pelegrina et al., 2015), in which children were asked to pay attention to a sequence of items to determine whether a stimulus presented on the screen matched a stimulus previously presented; (ii) the abstract shapes task, based on Davidson et al., (Davidson, Amso, Anderson, & Diamond, 2006), in which children were taught a rule for each stimulus; (iii) the WM span task, based on Petrides et al., (Petrides, 1995), in which children were required to select the stimulus that had not been presented in the previous set of stimuli. The math training (developed by Educamigos S.L.) consisted of a set of exercises to practice basic math skills.

### *WM Training Tasks*

In the *n*-back task, we manipulated a total of three variables, memory load, presentation time, and stimulus type, to create multiple difficulty levels. The WM load was modified by using three levels of complexity (1-, 2-, 3-back). The presentation time was either 500 ms (easy trials) or 1000 ms (difficult trials). Furthermore, we had two difficult levels within each stimulus type: alphanumeric stimuli (a small set with 5 elements and a big set with 10 elements), shapes (simple and complex shapes, such as squares and drawings without key patterns, respectively), words (an easy level with different semantic categories and a difficult level with identical semantic categories), and drawings at the same levels as the “words” stimuli).

As shown in Figure S2, the instructions appeared on the screen until the child was ready (s/he was required to press a bottom to continue), and a white screen with the word loading was then presented for 3 seconds. Each trial began with a white screen (500 or 1000 ms, randomly assigned), and the stimulus was then presented for 500 or 1000 ms depending on the level of difficulty. The child could respond in the 5000 ms from the time the stimulus first appeared on the screen. An audio feedback for each response with a duration of 500 ms was provided to the child (one sound for correct trials and another for incorrect trials). Each block included 3 trials that consisted of stimuli not presented previously, 10 trials that consisted of 6 stimuli not previously presented and 4 stimuli previously presented. This sequence was the same for all types of *n*-back tasks. A 95% correction rate on a block was considered a successful performance for that block, and the child advanced to the next level when s/he successfully completed two blocks.


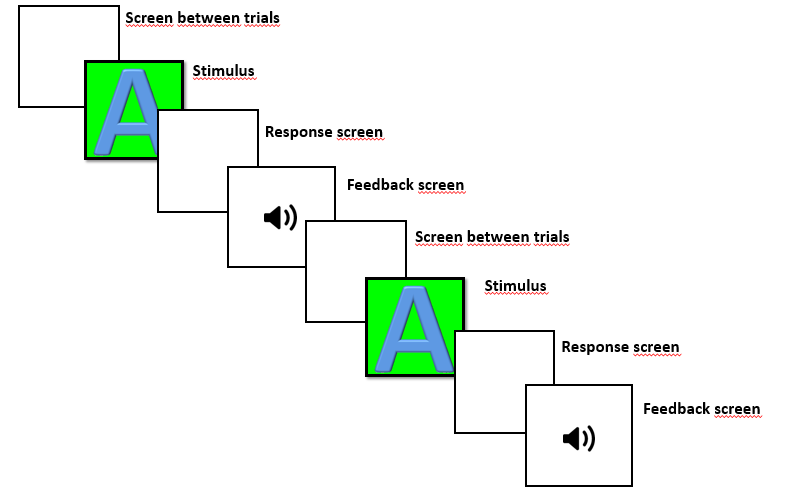


**Figure S2.** Example of a n-back trial.

In the abstract shapes task, children were taught a rule for each stimulus, i.e., press the right button for this stimulus, and press the left button for that stimulus. In this case, we manipulated two variables to create multiple difficulty levels: set size and time response. We had 4 set sizes ranging from the easiest to the most difficult level: 2 stimuli (where the child was required to press the right button for one stimulus and the left button for the other stimulus), 4 stimuli (in which the child was required to press the right button for two stimuli and the left button for the other two stimuli), and 6 stimuli (where the child had to press the right button for three stimuli and the left button for the other three stimuli). There was also a level with 8 stimuli, where the child had to press the right button for four stimuli and the left button for the other four stimuli, but no child reached this level.

Within each set size, 5 levels of difficulty were determined by the maximum time the child had to respond. In the easiest level, the child had 10 seconds to respond. We took into account the child’s average reaction time on the first block and estimated the maximum response times for the other levels using the following mathematical formula: Average + SD/2 (2nd difficulty level), average (3rd difficulty level), average – SD/2 (4th difficulty level), and average – SD (5th difficulty level).

The experimental design showed the following structure (see Figure S3): the instructions were presented on the screen until the child was ready (s/he had to press a bottom to continue), and a white screen with the word “loading” was presented for 3 seconds. Each trial began with a white screen (500 or 1000 ms, randomly assigned), and the stimulus appeared. The child had to respond within a specific time period that was dependent on the level of difficulty. On the same screen, the child received an audio feedback lasting 500 ms for each trial—one sound for correct trials and another sound for incorrect trials. Each block included 24 trials. A 95% correction rate on a block was considered a successful performance for that block, and the child was advanced to the next level when s/he successfully completed two blocks.

**
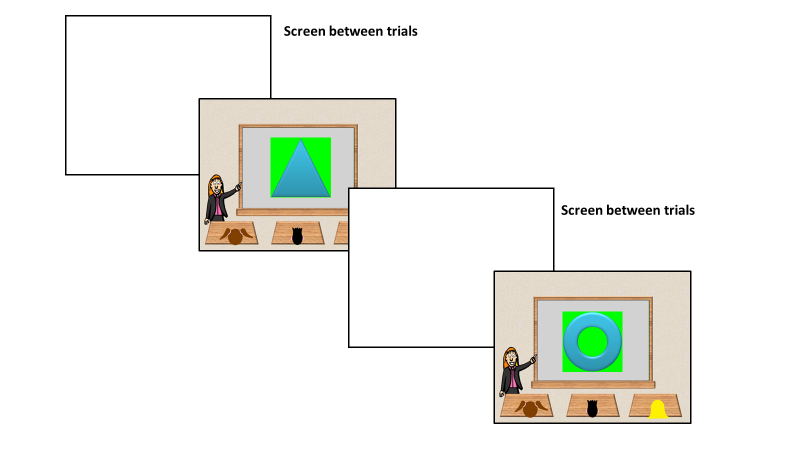
**

**Figure S3.** Example of an abstract shapes trial.

Finally, in the third task, a working memory span task, we manipulated 2 variables to create different levels of difficulty: set size and retention time. With respect to set size, from the easiest to the most difficult level, 3, 4, 5, and 6 stimuli were presented, including the target. The retention time, from when the set presentation was finished, and a question mark was presented (see Figure S4) to when the child replied, also had four levels of difficulty: 1000, 2000, 4000, and 8000 ms.

As in the other tasks, instructions were presented on the screen until the child was ready (s/he had to press a bottom to continue), and a white screen with the word “loading” appeared for 3 seconds. Each trial began with a white screen (500 or 1000 ms, randomly assigned) that preceded the presentation of the stimuli (500 ms), between which a white screen appeared (500 ms). The stimuli presentation, the time of which was dependent on the level of difficulty, ended with a question mark followed by a screen on which two stimuli appeared to which the child was required to reply (2000 ms). An audio feedback was presented once the child replied (500 ms, one sound for correct and another for incorrect responses). Each block included 20 trials. When the child reached 95% accuracy in a block, it was considered a successful performance, and the child passed to a higher level of difficulty when s/he successfully completed two blocks.

**
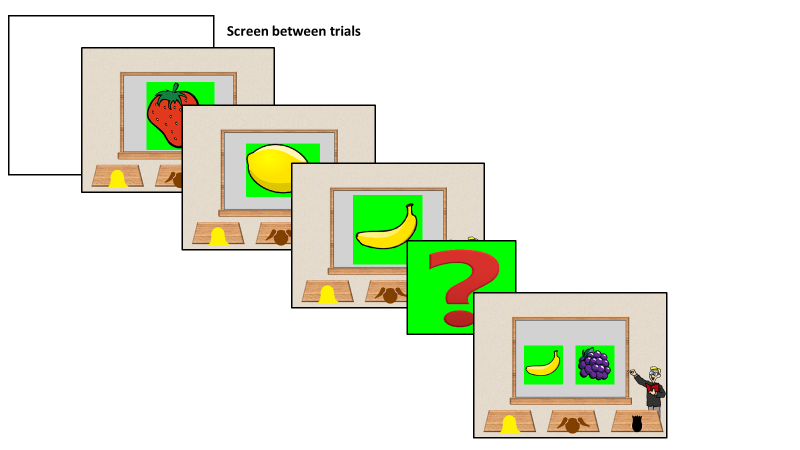
**

**Figure S4.** Example of a working memory span trial.

### *Math Training Tasks*

Math training (developed by Educamigos S.L.) included the following exercises:

1. *Rain of numbers*, in which the child was required to correctly solve the highest number of mathematical operations (additions, subtractions, divisions, and multiplications) within a two-minute time limit.

2*. Numerical series*, wherein the child had to complete the highest number of numerical sequences within a two-minute time limit.

3. *Day-to-day situations*, wherein the child was asked to solve the highest number of mathematical problems within a two-minute time limit.

4. *Digits discrimination*, in which the child was required to sort the highest number of digits in a list in ascending and decreasing order. At the same time, some digits were presented upside-down, and s/he had to identify the highest number of digits as soon as possible.

To complete the operations, the child was asked to complete the highest number of mathematical operations within a two-minute time limit.

## Behavioral data

## To avoid repetition with the Sánchez-Pérez et al. (2018) study, only tasks that reproduced significant differences between the two groups are referred to here.

## *Cognitive Skills*

## *Intelligence (IQ).* Children’s IQs were measured by the Spanish version of the Kaufman Brief Intelligence Test (K-Bit; Kaufman & Kaufman, 1990). This test provides an index of verbal intelligence that covers the knowledge of language, the formation of verbal concepts and a wealth of information. It also provides another index for non-verbal IQ, which requires non-verbal reasoning and flexible problem-solving skills.

## *Inhibition (go/nogo task).* Inhibition skills were measured by an adaptation of the task developed by Durston et al., Durston et al., 2002). Children were not only required to press a button on the joystick when a target animal (go trials, randomly extracted from a set of 10 animals) appeared but also to avoid responding when a specific non-target animal (a lion, no-go trials) appeared. The task began with a block of 10 practice trials, followed by 40 go trials. The participants completed an experimental block of 168 go/nogo trials. Thus, there were 14 nogo trials for each number of preceding go sequences, where the total number of go trials varied for the sequenced 1, 3 and 5 preceding go trials, i.e., 14, 42, and 70 go trials, respectively.

## *Academic Achievement*

## *Mathematical abilities.* To evaluate the children’s math abilities, the Spanish version of the Woodcock-Johnson III (WJ-III) Achievement battery was used (Woodcock, McGrew, & Mather, 2001). This battery includes the following math tasks/scales: Calculations, aimed to measure a child’s ability to perform simple mathematical computations (addition, subtraction, multiplication, and division); math fluency, which measures the ability to quickly solve simple calculations; applied problems, evaluating the ability to analyze and solve math problems; and quantitative concepts, which measures a student’s knowledge of mathematical concepts, symbols, and vocabulary. According to the Rasch measurement model (Rasch, 1960; Wright & Stone, 1979), the raw scores of each of these scales were transformed into W scores (Woodcock, 1978; Woodcock & Dahl, 1971). However, apart from these individual scales, the following W composite scores provided by the WJ-III were also considered: Broad math (including the calculations, math fluency, and applied problems scales); brief math (considering calculations and applied problems); math calculation skills (including calculations and math fluency); and (4) math reasoning (considering applied problems and quantitative concepts).

## *Reading abilities.* The children’s reading abilities were measured using five subtests from the PROLEC-R (Batería de Evaluación de los Procesos Lectores – Revisada; Reading Process Evaluation Battery-Revised; Cuetos, Rodríguez, Ruano, & Arribas, 2007). In the first subscale, called the name or sound of letters, the child was required to name or say the sound of 20 written letters. In the same-different subtest, the child was asked to inform whether the two words or pseudo-words presented were the same or different (half of the 20 pairs were the same, and the other half were different) to assess whether the child exhibited logographic reading. In the third subtest, word reading, which measures the letter recognition process, the child was required to read 40 words (20 of which were high-frequency appearing words and 20 of which were low-frequency appearing words). In the following subtest, pseudo-word reading, the child was also asked to read a total of 40 stimuli, pseudo-words, to assess their accuracy in identifying pseudo-words or non-existing words.

***Control variables***

Parents completed a questionnaire regarding their years of schooling (fathers and mothers) and their monthly family income in a scale from 1 (less than 750 Euros) to 6 (more than 3000 Euros). A socioeconomic status (SES) index was calculated by standardizing and averaging these three variables. Child’s gender was coded as (0) for girls or (1) for boys. Child’s age was reported in years at the beginning of this study.

**Statistical Analyses**

Given that one of the aims of this study was to reproduce the findings observed in the overarching study by Sánchez-Pérez et al. (Sánchez-Pérez et al., 2018) in the fMRI study subsample, we followed the same steps reported in that study. First, we ran independent *t*-tests to examine potential sociodemographic differences between the control and training groups at the baseline. Second, analyses of variance (ANOVAs) and independent *t*-tests were run to assess the gender and age effects on pre-score variables. Third, analyses of covariance (ANCOVAs) were computed to analyze whether the training group outperformed the control group in the cognitive and academic outcomes.

**References**

Cuetos, F., Rodríguez, B., Ruano, E., & Arribas, D. (2007). PROLEC-R. Batería de evaluación de los procesos lectores, revisada. *Madrid: TEA*.

Davidson, M. C., Amso, D., Anderson, L. C., & Diamond, A. (2006). Development of cognitive control and executive functions from 4 to 13 years: Evidence from manipulations of memory, inhibition, and task switching. *Neuropsychologia*, *44*(11), 2037–2078. https://doi.org/10.1016/j.neuropsychologia.2006.02.006

Durston, S., Thomas, K. M., Yang, Y., Uluğ, A. M., Zimmerman, R. D., & Casey, B. J. (2002). A neural basis for the development of inhibitory control. *Developmental Science*, *5*(4), F9–F16. https://doi.org/10.1111/1467-7687.00235

Kaufman, A. S., & Kaufman, N. L. (1990). *K-BIT: Kaufman brief intelligence test*. Minnesota: American Guidance Service.

Pelegrina, S., Lechuga, M. T., García-Madruga, J. A., Elosúa, M. R., Macizo, P., Carreiras, M., … Bajo, M. T. (2015). Normative data on the n-back task for children and young adolescents. *Frontiers in Psychology*, 1544. https://doi.org/10.3389/fpsyg.2015.01544

Petrides, M. (1995). Impairments on nonspatial self-ordered and externally ordered working memory tasks after lesions of the mid-dorsal part of the lateral frontal cortex in the monkey. *The Journal of Neuroscience: The Official Journal of the Society for Neuroscience*, *15*(1 Pt 1), 359-375.

Rasch, G. (1960). Probabilistic models for some intelligence and achievement tests. *Copenhagen: Danish Institute for Educational Research*.

Sánchez-Pérez, N., Castillo, A., López-López, J. A., Pina, V., Puga, J. L., Gonzalez-Salinas, … Fuentes, L. J. (2018). Computer-based Training in Math and Working Memory Improves Primary School Children’s Academic and Cognitive Competences: Behavioral Results. *Frontiers in Psychology*, *8*. https://doi.org/10.3389/fpsyg.2017.02327

Woodcock, R. W. (1978). *Development and standardization of the Woodcock-Johnson psycho-educational battery*. Hingham: MA: Teaching Resources.

Woodcock, R. W., & Dahl, M. N. (1971). *A common scale for the measurement of person ability and test item difficulty (AGS Paper No.10)*. Circle Pines, MN: American Guidance Service.

Woodcock, R. W., McGrew, K. S., & Mather, N. (2001). *Woodcock-Johnson® III NU Tests of Achievement*. Itasca, IL: Riverside.

Wright, B. D., & Stone, M. H. (1979). *Best Test Design. Rasch Measurement.* Chicago: Mesa Press.
